# Resistance is not futile: a systematic review of the benefits, mechanisms and safety of resistance training in people with heart failure

**DOI:** 10.1007/s10741-024-10402-0

**Published:** 2024-04-15

**Authors:** Bradley A. Morris, Ronak Sinaei, Neil A. Smart

**Affiliations:** University of New England, Armidale, NSW 2350 Australia

**Keywords:** Cardiomyopathy, Strength training, Resistance training

## Abstract

Exercise offers many physical and health benefits to people with heart failure (CHF), but aerobic training (AT) predominates published literature. Resistance training (RT) provides additional and complementary health benefits to AT in people with CHF; we aimed to elucidate specific health benefits accrued, the mechanism of effect and safety of RT. We conducted a systematic search for RT randomised, controlled trials in people with CHF, up until August 30, 2023. RT offers several benefits including improved physical function (peak VO_2_ and 6MWD), quality of life, cardiac systolic and diastolic function, endothelial blood vessel function, muscle strength, anti-inflammatory muscle markers, appetite and serious event rates. RT is beneficial and improves peak VO_2_ and 6MWD, partly restores normal muscle fibre profile and decreases inflammation. In turn this leads to a reduced risk or impact of sarcopenia/cachexia via effect on appetite. The positive impact on quality of life and performance of activities of daily living is related to improved function, which in turn improves prognosis. RT appears to be safe with only one serious event reported and no deaths. Nevertheless, few events reported to date limit robust analysis. RT appears to be safe and offers health benefits to people with CHF. RT modifies the adverse muscle phenotype profile present in people with CHF and it appears safe. Starting slowly with RT and increasing load to 80% of 1 repetition maximum (RM) appears to offer optimal benefit.

## Introduction

Most people with congestive heart failure (CHF) are severely de-conditioned so any exercise training delivery is likely to accrue benefits in both physical and mental health [1]. Resistance training (RT) offers many advantages to people with CHF, perhaps the most notable is restoration of muscle strength due to changes in fibre type and function. Although aerobic training is often the cornerstone of exercise rehabilitation in people with CHF, this form of training may not be optimal for altering muscle fibre deterioration in older people or those with CHF. As we age, RT is required to prevent a shift towards type II muscle fibre size decreases, even in healthy people [2]. This type II muscle fibre shift is even more pronounced in people with CHF, but in these patients there is also a shift towards reductions in the number and size of type I fibres [2]. These muscle fibre type changes may be driven by inflammatory mediators detected in the muscle but not systemic circulation [3].

Between 2007 and 2016, there may have been hesitancy around the use of RT in people with CHF especially in those with very low left ventricular ejection fractions (LVEF%). This may be explained by Haykowsky’s 2007 meta-analysis in people with CHF that suggested whilst AT was beneficial for LVEF, RT may lead to a reduction in LVEF of around 4.5% [4]. There were, however some limitations to this finding, namely it was based upon one study [5] that pre-dated modern imaging techniques and the study size was only 25 participants. In 2016, an updated meta-analysis, which included three new RT studies, abrogated these concerns [6]. Both fibre type changes and reduced LVEF% may contribute to lower peak VO_2_ in people with CHF. Peak VO_2_ remains strongly prognostic in people with CHF [7].

In 2021, we updated our 2016 meta-analysis and presented novel findings suggesting RT raised both peak VO_2_ and six-minute walk distance (6MWD) independently of LVEF% change [8]. Whilst the number of randomised, controlled studies of RT is relatively small compared to AT, there is evidence of cardiovascular benefit in people with CHF. There also exists, in the published literature, evidence that RT also provides benefits in terms of quality of life (QoL) [1], non-cardiac muscle strength [9], endothelial function [10], heart rate variability [11], insulin sensitivity [12], sleep quality [13] and serum BNP [14, 15]. These findings give rise to the options that RT remains perhaps under-utilised in people with CHF and the mechanisms of benefit are not well understood, although peak VO_2_ has been shown to improve prognosis [7]. Further comprehensive safety data for the use of RT in people with CHF are yet to be published.

This work aimed to provide a systematic review of the benefits and safety from published randomised, controlled trials of RT in people with CHF. Further, we also considered data from non-randomised controlled trials to identify mechanisms of effect from RT in people with CHF.

## Methods

### Search strategy

We began with our study by Fisher et al. which was a 2022 meta-analysis of 17 randomised, controlled trials (RCTs) on resistance training (RT) in people with heart failure [8]. Our 2022 work was a pooled analysis of 13 RCTs compared RT to sedentary control, 2 RCTs compared RT to aerobic training (AT) and 2 duplicate publications that provided some additional data beyond the primary publication.

We updated the literature search August 2023. No new RCTs of RT in people with CHF were found, but several mechanistic papers proved useful and formed part of this body of work. We conducted the systematic literature search in PubMed, Web of Science and the Cochrane Library of Controlled Trials up until 30 September 2023. The search strategy included key words related to congestive heart failure and exercise training and related MeSH terms. This was supplemented by manually searching reference lists from systematic reviews and eligible studies for additional works. The strategy for database searches is documented here [8]. This search strategy was repeated for the additional data bases used: Web of Science and Cochrane Library of Controlled Trials.

### Inclusion and exclusion criteria

Two authors (RS and NAS) independently assessed all identified articles for eligibility and joint consultation was used to resolve any disagreement.

### Included studies

We included RCTs conducted on adult humans (over 18 years) that reported change in listed outcomes after exercise training. RT studies were considered provided the intervention was for a minimum of 3 weeks duration. Crossover studies were only excluded if the washout period was less than 2 weeks. There was no language restriction. We also drew upon comparative data on AT from several other reviews and meta-analyses [11, 16–21].

### Excluded studies

Identified studies not reporting any of the required outcomes were excluded. Studies with participants with any medical condition that impaired RT participation were excluded.

### Comparisons

Included studies compared RT intervention group(s) to a non-exposed, matched health status, control (usual care) group or a sham intervention group.

### Outcome measures

The primary outcome measures were change in peak VO_2_, 6MWD, muscle fibre type, area and sphericity, cardiac systolic and diastolic function, forearm blood flow, endothelial function, heart rate variability, systemic or intramuscular inflammation, quality of life, energy intake and appetite, adverse events and safety of RT.

### Data extraction

From each included study, we extracted the first author’s name, year of publication, country, study design, type of study population and participants’ baseline characteristics (including age, gender, number of participants and resting BP). In addition, the characteristics of training interventions (i.e. exercise program delivery venue and method, type of exercise, intensity, duration and frequency of the protocol) and the mean change and standard deviation of the desired outcome variables were recorded. Data extraction was conducted independently by two authors (RS and NAS) using a predesigned Excel data extraction sheet.

### Statistical analyses

Data sets were organised and descriptive analyses performed using Excel 2016 for all included studies. As meta-analyses were completed for our previous works [6, 8], we calculated weighted means and standard deviations in STATA V.18 [22]. Weighted means were used for most outcome measures. We also estimated the minimal clinically important difference (MCID) for some outcomes using the change in standard deviation divided by 2 [23].

### Risk of bias

Study quality and reporting for each study was evaluated utilising the TESTEX tool [24].

## Results

Our search found the original 17 randomised, controlled trials from our earlier publication and one new trial. Of the 18 trials, 14 compared RT to control, 2 compared RT to AT and 2 were duplicate publications (see Table 1). We also considered other types of papers to provide comparative data of other types of exercise (i.e. aerobic) so that RT could be benchmarked against other types of exercise for outcomes such as safety. We have listed sources of comparative data in the relevant sections below.

**Table 1:** Included studies table

**Study**	**RT/Con/AT**	**Inclusion/exclusion**	**Outcome(s)**
Cider et al. [25]	12/10	CHF diagnosis > 1 year, NYHA class 2A–3B	Peak VO_2_, 6MWD
Feiersen et al. [26]	45/15	Inclusion: 40–70 years, NYHA II–III, LVEF < 35% Exclusion: NYHA class IV, malignant ventricular arrhythmias, renal dysfunction, stroke, COPD and orthopaedic limitations	Peak VO_2_, LVEF
Gielen et al. [3]	10/10	Male < 70 years with CHF, NYHA II–III, LVEF < 40%	Peak VO_2_, TNF-alpha, IL-6, adverse events
Groennebaek et al. [27]	12/12/12RIC	18 to 80 years, LVEF ≤ 45%, NYHA I–III	6MWD, blood flow, fibre type, BNP, diastolic function, QoL Minnesota
Grosse et al. [28]	14/13		Peak VO_2_
Jakovjelic et al. [29]	21/0/AT 11	LVEF < 40%, NYHA II–III	Peak VO2
Koch et al. [5]	21/11	LVEF < 40%, NYHA II–III	LVEF
Lan et al. [30]	12/12/12 AT	LVEF < 50%	Peak VO_2_, LVEF, diastolic function
Levinger et al. [9]	8/7	CHF diagnosis	Peak VO_2_, QoL Minnesota
Maiorana et al. [17]	24/12/AT12	LVEF < 50%, NYHA I–III	Peak VO_2_, LVEF, endothelial function
Munch et al. [31]	14/0/AT12	LVEF < 40%, NYHA I–III	6MWD, LVEF, endothelial function, TNF-alpha, QoL Minnesota
Palevo et al. [32]	10/6	LVEF < 40%, NYHA I–III	6MWD, LVEF
Pu et al. [33]	9/7	Community-dwelling women > 64 years, LVEF < 45%, NYHA I–III	Peak VO_2_, 6MWD, LVEF, diastolic Fn., E/A ratio, fibre type
Redwine et al. [34]	22/23	CHF diagnosis, > 40 years	Peak VO_2_, 6MWD
Sadek et al. [35]	8/8	LVEF < 45%, NYHA II–III	6MWD, LVEF
Selig et al. [11]	19/20	LVEF < 40%, NYHA II–IV, no aortic stenosis	Peak VO_2_, forearm blood flow, HRV
Tyni-Lenne et al. [36]	16/8	LVEF < 40%, NYHA II–III	Peak VO_2_, 6MWD
Xu et al. [37]	32/28	NYHA II–III	6MWD
Total	300/202/47		

*RT* resistance training, *AT* aerobic training, *Con* control, *RIC* remote ischemic conditioning, *6MWD* six-minute walk distance, *LVEF* left ventricular ejection fraction, *HRV* heart rate variability, *QoL* quality of life, *Minnesota* Minnesota living with heart failure questionnaire score, *NYHA* New York Heart Association, *IL-6* Interleukin-6

### Physical fitness

**Fig. 1:**
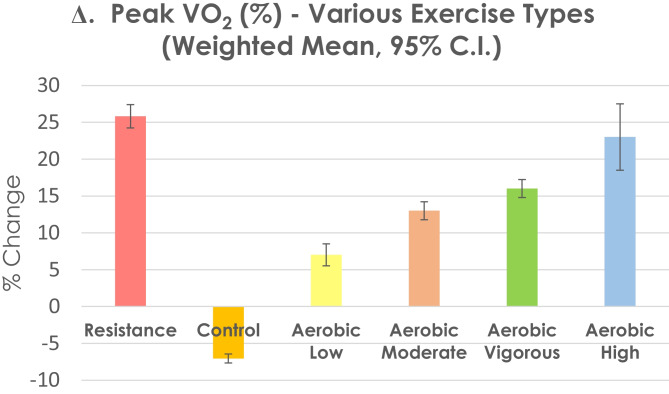
Weighted % change in peak VO_2_ following resistance training (RT) in people with heart failure

**Fig. 2:**
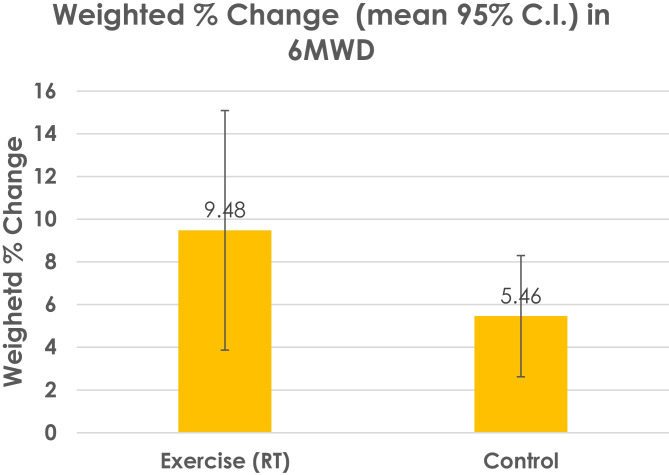
Weighted % change in 6-min walk distance (6MWD) following resistance training (RT) in people with heart failure

We compared (see Fig. 1) the percentage change in peak VO_2_ between RT and AT at various intensities, and also sedentary control, using comparative data from Ismail et al. [38]. We found RT produced a similar peak VO_2_ change of 25.5 ± 1.65 ml·kg·min^−1^ to that previously observed with aerobic exercise at high intensity which was significantly greater than peak VO_2_ changes reported following vigorous, moderate or low intensity [38]. Non-exercising control participants’ peak VO_2_ decreased by 7%. We found a 9.5 ± 4.8% improvement in 6-min walk distance following RT, although control participants improved 5.5% (see Fig. 2). Further, we found the changes in both peak VO_2_ to be above the minimal clinically important difference (MCID) of the SD (1.65 ml·kg·min^−1^/2) at 0.825 ml/kg·min^−1^. We calculated the MCID or 6MWD to be 39 m, which is almost identical to previous work that demonstrated that a 6MWD increase of around 40 m is the MCID threshold for change in people with CHF [39].

### Cardiac function

**Fig. 3:**
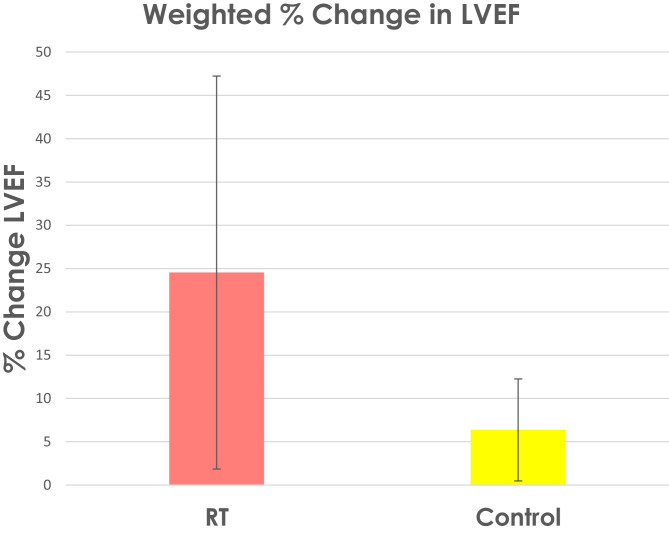
Weighted change in LVEF systolic function

Pooled data from 7 studies showed systolic function, measured by LVEF, improved with RT by 24.8 ± 10.9% whilst non-exercising control participants with CHF improved by 6.1 ± 4.3% (see Fig. 3).

**Fig. 4:**
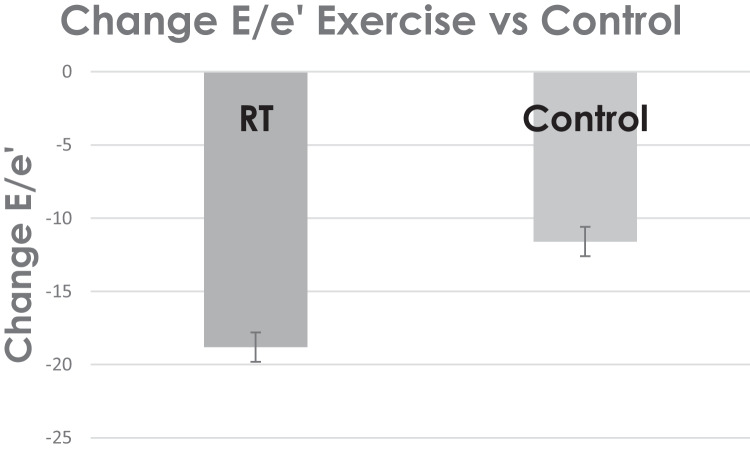
Weighted change in diastolic function (E/e’)

Data from one study suggested diastolic function, as measured by E/e’, decreased and thereby improved by − 18.8 ± 7.7% and by − 11.8 ± 6.5% in non-exercising control participants with CHF (see Fig. 4). Improved cardiac function is closely related to lower left atrial volume index and prevention of raised LV mass index, both indices are strong predictors of cardiac events [18].

### Quality of life

**Fig. 5:**
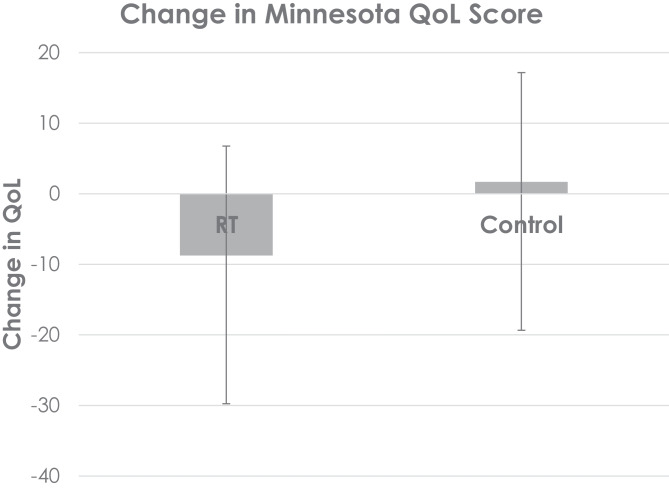
Weighted percentage change in Minnesota living with heart failure score

Only two studies reported change in Minnesota living with heart failure questionnaire scores (MLWHFQ) in exercise versus control groups. The MLWHFQ is a measure of quality of life, which was improved (lower score) by almost 8.75 points (30%) in people who undertook RT. In contrast, there was a small reduction (higher score) in quality of life scores in the control group (see Fig. 5).

### RT and inflammatory muscle markers of CHF

Work by Larsen showed significantly greater area of type I fibres in healthy people versus those with CHF, whilst type IIA fibres were more common in CHF. Thickness of type IIA and IIB was significantly greater in healthy people versus those with CHF [2]. Larsen also reported greater sphericity of type 2A muscle fibres in people with CHF versus healthy [2]. Further, two studies Gielen [3] and Larsen [2] showed that intramuscular levels of TNF-alpha were significantly higher in people with CHF versus those who are healthy. Larsen further showed that IL-1, IL-6 and the inducible form of nitric oxide synthase mRNA were significantly higher in people with CHF versus healthy [2]. Skeletal muscle IGF-1 levels are also increased with exercise to induce protein synthesis [3]. Exercise has anti-inflammatory/antioxidant effects and likely combats cachexia [19, 40].

### Blood vessel function and heart rate variability

Previous work has identified that exercise has a systemic impact on remodelling of conduit arteries in humans and that RT may be advantageous in subjects with chronic heart failure in this regard [17]. Further, both forearm blood flow [17] and heart rate variability are improved in people with heart failure following RT, with evidenced by a reduced ratio low- to high-frequency spectral power [11].

### Appetite, energy expenditure and cardiac cachexia

Andreae showed daily step counts were positively associated with better appetite in people with CHF which may abrogate adverse prognosis due to cardiac cachexia [20]. After 18-month follow-up, Andreae showed that people with CHF who were more active retained their physical function. The obesity paradox [41] explains the lower mortality rates observed in obese/overweight people with CHF due to cardiac cachexia. Both aerobic and RT exercise have been previously shown to improve appetite and energy intake in people with chronic kidney disease [21].

### Safety of RT in CHF

Based upon the included studies of this review, no deaths during RT have been reported in people with CHF in over 8000 patient-hours of exercise. The only event of note is a single episode of ventricular arrhythmia reported in one study in a person undertaking RT [3].

All that can be concluded from existing data is that limited safety data currently exists for RT in CHF as most studies are small and < 12 weeks. Currently there exists an insufficient number of RT related events that precludes a meaningful statistical analysis. We conducted a power calculation that indicated around 200 events (deaths) would be required to detect a statistically significant difference for safety in RT versus control groups.

Other germane observations around event data collection are that there is clearly heterogenous and irregular event data collection. Some studies only report events that occur during RT exercise sessions; other studies collect and report event data during entire study duration period when people may be sleeping or resting. Another observation to make is that the terminology and definitions vary by study, for example the threshold for what constitutes a serious event is not standardised.

**Fig. 6:**
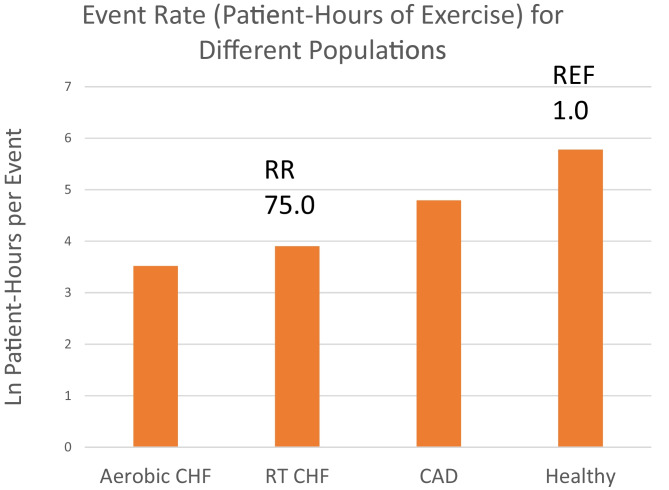
Event rate per patient-hours of exercise for different populations

We cannot be unequivocally certain if RT is safe or unsafe without more event data, but no published evidence of serious events to date suggests RT is safe. In comparison, AT has an event rate of 1 in 3300 patient-hours for CHF [42]. The event rate for coronary artery disease is 1 in 62,000 patient-hours of AT and in a healthy population the event rate is 1 in 600,000 [43] (see Fig. 6). The literature on RT and CHF currently has data from 15 randomised, controlled trials (RCTs) which is relatively scarce compared to the 150 RCTs on AT and CHF.

### Markers of event risk

During RT the rate pressure product (RPP) is possibly around 5% lower than in AT at moderate [44] intensity (55–70% age-predicted max HR or 40–60% peak VO_2_) and the intermittent nature of RT likely to reduce event risk. If one approximates heart rate (HR) and systolic blood pressure (SBP) during AT and RT as follows:

Aerobic Training: HR 130 beats·min^−1^ × (peak) SBP 160 mmHg = RPP 20,800.

Resistance Training: HR 110 × (peak) SBP 160 = RPP 17,600.

The slightly lower RPP during RT is likely to translate into a safer exercise modality than AT, especially when one compares the sustained HR and BP levels with the shorter peaks observed with RT. Additional evidence of cardiovascular event risk benefit lies with the improved heart rate variability (HRV) reported following a program of RT with the ratio of low-to-high-frequency spectral power reduced by 44% [11].

### Study quality

Table 2 shows study quality and reporting for each study utilising the TESTEX tool.

**Table 2:** TESTEX evaluation of study quality and reporting

**Study ref.**	**Eligibility criteria specified**	**Randomisation specified**	**Allocation concealed**	**Groups similar at baseline**	**Blinding of assessors**	Outcome measures in > 85% participants*	**Intention to treat analysis**	Between-group statistical comparisons reported**	**Point measures and measures of variability reported**	**Activity monitoring in control group**	**Relative exercise intensity reviewed**	**Exercise volume and energy expended**	**Overall TESTEX**
Cider et al. [25]	1	1	0	1	0	2/3	0	2/2	1	0	1	1	10
Feiereisen et al. [26]	1	0	0	1	0	3/3	0	2/2	1	0	1	1	10
Gielen et al. [3]	1	0	0	1	1	2/3	0	2/2	1	0	0	1	9
Groennebaek et al. [27]	1	1	0	1	1	3/3	0	2/2	1	0	1	0	11
Grosse et al. [28]	1	1	0	1	0	3/3	0	2/2	1	0	1	1	11
Jakovljevic et al. [29]	1	0	0	1	0	1/3	0	2/2	1	0	1	1	8
Koch et al. [5]	1	0	0	1	0	2/3	0	2/2	1	0	1	1	9
Lan* et al. [30]	1	0	0	0	1	2/3	0	2/2	1	0	1	1	9
Levinger et al. [9, 45]	1	0	0	1	0	3/3	0	2/2	1	1	1	1	11
Maiorana* et al. [17]	1	0	0	1	1	3/3	0	2/2	1	0	1	1	11
Munch et al. [31]	1	1	0	1	1	3/3	0	2/2	1	0	1	1	12
Palevo et al. [32]	1	0	0	1	1	3/3	0	2/2	1	0	1	0	10
Pu et al. [33]	1	1	1	1	1	3/3	1	2/2	1	0	1	1	14
Redwine et al. [34]	1	1	1	1	1	3/3	0	2/2	1	1	0	0	12
Sadek et al. [35]	1	1	0	1	0	3/3	0	2/2	1	0	1	1	11
Selig et al. [11]	1	1	0	1	0	3/3	0	2/2	1	1	1	1	12
Tyni-Lenne et al. [36]	1	1	0	1	0	2/3	1	2/2	1	0	1	1	11
Xu et al. [37]	1	0	0	0	0	1/3	0	2/2	1	0	0	0	5
Totals													Median 11

TESTEX total out of 15 points. *Three points possible, **two points possible. A score of zero (0) was allocated if it was unclear. If ITT was not specifically mentioned, but it was noted that no participants withdrew, and all analysed 1 point was awarded

The median TESTEX score was 11 out of 15 which previous work indicates overall good study quality [46]. Allocation concealment, intention to treat (ITT) analysis and physical activity monitoring in the control group were the only TESTEX criteria performed on less than 50% of the 24 included studies. Allocation concealment is very difficult in exercise studies and ITT is only relevant if > 15% of participants withdraw prematurely from the study.

### Risk of bias

The overall risk of bias was low as median TESTEX score indicated overall good study quality. One study scores 5, but this relatively low score was attributable to the study being published in Chinese and an English version would have certainly yielded a higher score. One study scored 8 and three scored 9; the other 19 studies scored 10 or above, confirming generally low risk of bias.

## Discussion

### The need for RT for people with CHF

People with CHF are severely deconditioned [47], so there is large potential for improved peak VO_2_. Intuitively any form of exercise training is likely to benefit peak VO_2_. In contrast, in a person with CHF no regular exercise training or physical activity will lead to further deterioration in peak VO_2_ and this is often linked with sarcopenia and cachexia [15, 40]. As RT and AT appear to provide benefit via some shared, and some different, physiological pathways, a combination of RT and AT is likely to be optimal [48]. The fibre shift observed in people with CHF will, intuitively, require new type II fibres to possess oxidative capacity [49]. In turn this suggests a combined RT and AT training prescription is optimal.

### RT exercise prescription recommendations

#### Previous and current general RT prescription recommendations for CHF

Meyer [50] previously stated that dynamic RT exercise is well tolerated in chronic stable CHF if:

1. Initial contraction intensity is low.
2. Small muscle groups are involved.
3. Work phases are kept short.
4. Small number of repetitions per set is performed.
5. Work/rest ratio is > or = 1:2.

Meyer also suggested that with tolerance, contraction intensity can be increased [50]. Meyer proposed that following 12 weeks of RT, maximal strength could be improved by 15 to 50% [50]. Recent work by Paluch et al. [13] made recommendations for resistance training prescription for high-risk cardiovascular disease patients which included:

1. Intensity = <40% of 1RM
2. Number of repetitions = 15–20
3. Weekly frequency = ≥2 days per week
4. Planned rest days between sessions
5. Muscle adaptation = endurance
6. Resistance training modes = bodyweight, bands, machines and free weights

There appears to be some discrepancy between Meyer’s recommendations and those by Paluch and others. Meyer argued for a small number of repetitions to be performed, whereas Paluch et al. argued for 15–20 repetitions.

Most CHF RT studies have used less than 80% of 1RM which produced a standardised mean difference (SMD) of 0.76 [8]. Yet in 2005 Levinger et al. used a training intensity of 80% of 1RM or greater and their results produced an SMD of 1.07 [9], although overlap in measures of variance mean these SMDs were not significantly different. This finding raises a question about the current intensity guidelines and if they are too conservative? Could better functional outcomes be gained for CHF patients if they were provided with a periodised program that guided them towards using higher percentages of 1RM.

A recent systematic review and network meta-analysis by Chen and others, asked if ‘moderate resistance training is adequate for older adults with sarcopenia?’ [51] as sarcopenia is a common comorbidity for people with CHF [52]. Chen et al. stratified training intensity into three categories via the use of a description (light to moderate, moderate and moderate to vigorous), percentage of 1RM ranges (< 49%, 50–69% and 70–84%) and RPE ranges (using both a modified Borg scale of 1–10 and a traditional Borg scale of 6–20) [51]. This review concluded that moderate to vigorous (70–84% of 1RM, modified Borg RPE 7–8 or traditional Borg 14–17) intensity resistance training yielded greater muscle mass, lower extremity strength and physical performance than less intense training options [51]. The finding that higher intensity resistance training offers greater benefits to people with CHF [9] and concurrent sarcopenia [51] perhaps warrants changes to the current recommendations. We would perhaps add that RT should be complemented by AT as newly developed muscle fibres will require adequate O_2_ delivery. Further, as people with HF and preserved ejection fraction (HFpEF) tend to be older and or frail than those with reduced ejection fraction (HFrEF), we recommend early RT in those with HFpEF [47].

#### Specific RT considerations

Consideration needs to be given to program design with particular focus on training intensity and its classification. A patient’s understanding of training intensity is very important as training at the wrong intensity may lead to poor outcomes and excessive fatigue. Yet there are strategies available to increase a patient’s understanding of training intensity and mitigate the fatigue associated with resistance training whilst still reaping the benefits of increasing muscular strength. Often in people with chronic disease, a submaximal or non-failure resistance training protocol could be employed to reduce the fatigue associated with training to repetition failure, as non-failure training has been shown to be similarly effective for increasing strength as training to repetition failure [53] whilst minimising resistance training related fatigue and lowering training session intensity. To help patients understand different training intensities, a rate of perceived exertion (RPE) scale can be used, such as the OMNI-Resistance Exercise Scale [54–56]. However, another RPE scale, which has also been designed for resistance training, allows for patients to understand training intensities and non-failure training as the scale incorporates repetitions in reserve (RIR) [57]. The use of RIR can allow for individuals to autoregulate training intensity [57, 58]. RIR provides an overview of the Resistance Exercise-Specific RPE Scale and its relationship with RIR. For example, an RPE score on this scale of 8 out of 10 would indicate that 2 more repetitions could be completed at the end of a set (Table 3).

**Table 3:** Resistance Exercise-Specific RPE Scale with repetitions in reserve

**Rating**	**Rate of perceived exertion**
10	Maximum effort
9.5	No further repetitions, but could increase load
9.0	1 repetition remaining
8.5	1–2 repetitions remaining
8	2 repetitions remaining
7.5	2–3 repetitions remaining
7.0	3 repetitions remaining
5.0–6.0	4–6 repetitions remaining
3.0–4.0	Light effort
1.0–2.0	Little to no effort

Resistance Exercise-Specific RPE Scale designed by Zourdos et al. [57].

An RPE scale incorporating RIR and therefore a non-failure protocol allows for the overall intensity of the session to be adjusted or autoregulated by the patient. For instance, a patient could be programmed to complete a set on the leg press machine at 70% of 1RM with 2–5 repetitions in reserve allowing for autoregulation by the client.

Some clinical exercise physiologists may prefer to avoid 1RM testing as they believe it elevates injury risk. Yet, there is a large amount of evidence supporting the use and safety of 1RM testing in healthy populations [59], with emerging evidence of its reliability and safety for use with CHF patients [60]. However, 1RM testing is not necessary as intensity can be determined via the use of RPE scales [51, 54].

## Conclusions

RT is beneficial and improves peak VO_2_ and partly restores normal muscle fibre profile and decreases inflammation. RT at higher percentages of 1RM such as ≥ 80% appears to provide optimal improvements in strength and function and in turn this leads to a reduced risk or impact of sarcopenia/cachexia via effect on appetite. The positive impact on activities of daily living is related to improved peak VO_2_ which in turn improves prognosis. RT appears to be safe as there has been only one serious event reported and rate pressure product (RPP) during RT is lower than for aerobic exercise. Only one event has been reported in > 8000 patient-hours to date.

## Data Availability

The data was a pooled analysis of studies that are publicly available so anyone can extract this data of their own accord.
